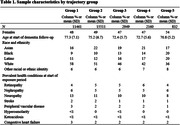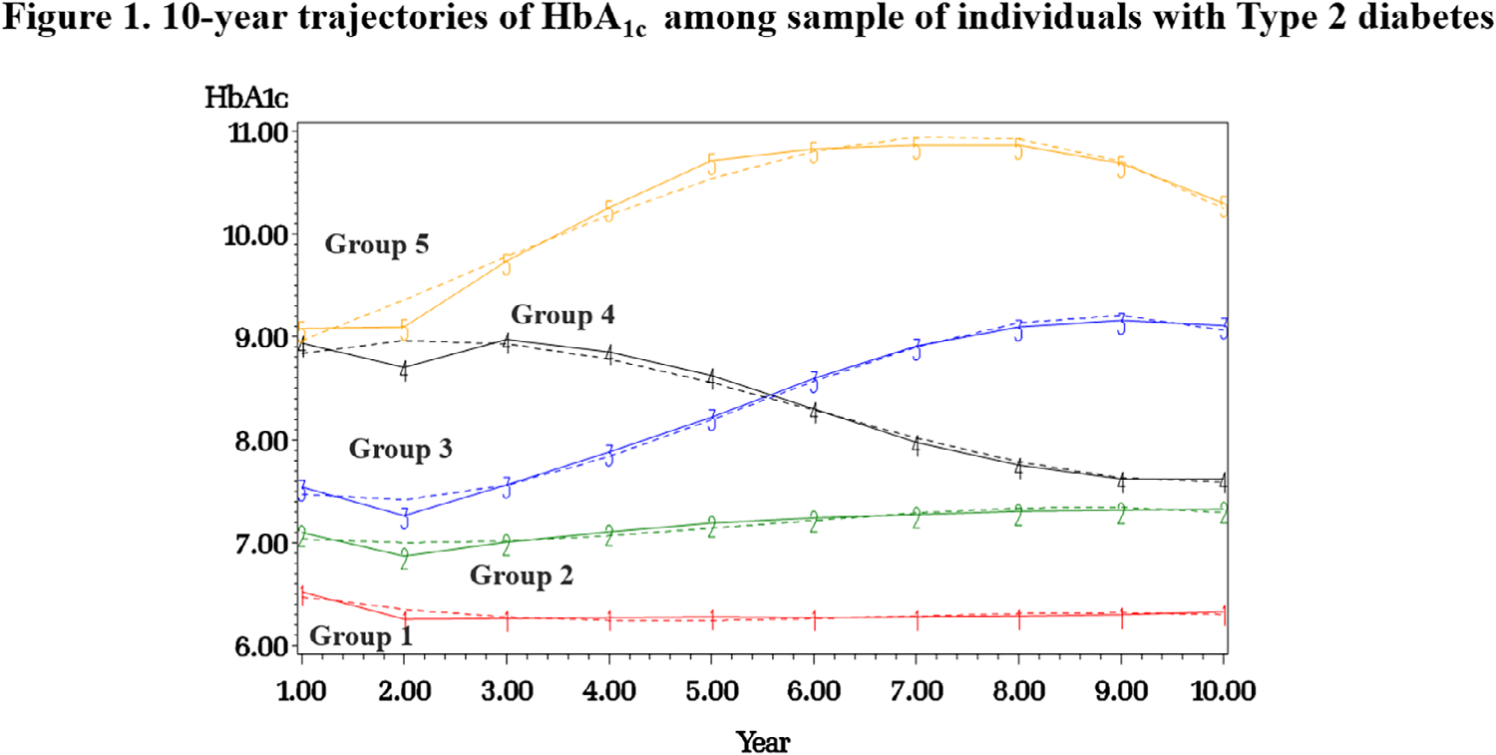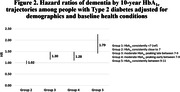# Ten Year Patterns of Change in HbA1C and Risk of Dementia in a Diverse Cohort of Adults with Type 2 Diabetes

**DOI:** 10.1002/alz.093385

**Published:** 2025-01-09

**Authors:** Rachel A. Whitmer, Chris Moran, Brianna Costales, Ai‐Lin Tsai, Mary E Lacy, Andrew Karter, Paola Gilsanz

**Affiliations:** ^1^ University of California, Davis, Davis, CA USA; ^2^ Monash University, Melbourne, VIC Australia; ^3^ Alfred Health, Melbourne Australia; ^4^ Peninsula Clinical School, Central Clinical School, Monash University, Melbourne Australia; ^5^ Kaiser Permanente Division of Research, Oakland, CA USA; ^6^ University of Kentucky, Lexington, KY USA; ^7^ Kaiser Permanente Northern California Division of Research, Oakland, CA USA

## Abstract

**Background:**

Though high HbA_1c_ (marker for glycemic control) is associated with increased dementia risk, the influence of longitudinal changes in HbA_1c_ is unclear. We examined the association between 10‐year trajectories of HbA_1c_ and dementia risk in a large, diverse sample of people in Northern California.

**Method:**

In a cohort of 32, 914 patients with Type 2 diabetes aged ≥ 50 years, we obtained repeated measures of HbA_1c_, dementia diagnoses, and comorbidities from electronic medical records (EMR) from 1996‐20021. We identified 5 latent trajectories of yearly mean HbA_1c_ over a 10‐year period (Figure 1): Group 1‐HbA_1c_consistently <7; Group 2‐ HbA_1c_ consistently close to 7; Group 3‐moderate HbA_1c_ peaking late between 7‐9; Group 4‐moderate HbA_1c_ peaking early between 7‐9; Group 5‐ HbA_1c_ consistently between 9‐11. Cox proportional hazards models estimated the association between HbA_1c_ trajectories and incident dementia ≥5 years after the end of the exposure period adjusting for age (as timescale) and prevalent health conditions (Table 1) at the beginning of the trajectory period. Stratified models examined possible effect modification by sex or race/ethnicity.

**Result:**

Approximately half of the sample were women (Table 1). The most common trajectory was Group 2‐consistently close to 7 (47%), followed by Group 1‐consistently <7 (35%), Group 3‐moderate peaking late (9%), Group 4‐moderate peaking early (7%), and Group 5‐ consistently high (3%). Compared to Group 1, Groups 3‐5 had elevated risk of dementia (Figure 2); risk was comparable in Groups 3 and 4 (HR_Group3_ = 1.30 (1.14, 1.48) and HR_Group4_ = 1.28 (1.10, 1.49)) and was highest in Group 5 (HR = 1.78 (1.42, 2.26). Group 2 was not at elevated dementia risk compared to Group 1 (HR = 1.02 (0.94, 1.09). These associations did not vary by sex or race/ethnicity.

**Conclusion:**

Over a 10‐ year period, consistently elevated HbA_1c_ levels ≥ 9 are associated with an 80% elevated risk of dementia compared to those with a trajectory of HbA_1c_ 6‐7. Those whose trajectory included HbA_1c_ levels of 8‐9 either early (Group 4) or late (Group 3) in the10‐year period had approximately 30% increased risk. These results suggest that there are long‐term consequences of elevated HbA_1c_ even if levels subsequently decrease.